# Clinical measures, radiomics, and genomics offer synergistic value in AI-based prediction of overall survival in patients with glioblastoma

**DOI:** 10.1038/s41598-022-12699-z

**Published:** 2022-05-24

**Authors:** Anahita Fathi Kazerooni, Sanjay Saxena, Erik Toorens, Danni Tu, Vishnu Bashyam, Hamed Akbari, Elizabeth Mamourian, Chiharu Sako, Costas Koumenis, Ioannis Verginadis, Ragini Verma, Russell T. Shinohara, Arati S. Desai, Robert A. Lustig, Steven Brem, Suyash Mohan, Stephen J. Bagley, Tapan Ganguly, Donald M. O’Rourke, Spyridon Bakas, MacLean P. Nasrallah, Christos Davatzikos

**Affiliations:** 1 Center for Biomedical Image Computing and Analytics (CBICA), University of Pennsylvania, 3700 Hamilton Walk, 7th floor, Philadelphia, PA 19104 USA; 2 Department of Radiology, Perelman School of Medicine, University of Pennsylvania, Philadelphia, PA USA; 3 Penn Genomic Analysis Core, Perelman School of Medicine, University of Pennsylvania, Philadelphia, PA USA; 4 Penn Statistics in Imaging and Visualization (PennSIVE) Center, Department of Biostatistics, Epidemiology, and Informatics, Perelman School of Medicine, University of Pennsylvania, Philadelphia, PA USA; 5 Department of Radiation Oncology, Perelman School of Medicine, University of Pennsylvania, Philadelphia, PA USA; 6 Abramson Cancer Center, Perelman School of Medicine, University of Pennsylvania, Philadelphia, PA USA; 7 Department of Neurosurgery, Perelman School of Medicine at the University of Pennsylvania, Philadelphia, PA USA; 8 Glioblastoma Translational Center of Excellence, Abramson Cancer Center, University of Pennsylvania, Philadelphia, PA USA; 9 Department of Pathology and Laboratory Medicine, Perelman School of Medicine, University of Pennsylvania, Philadelphia, PA USA

**Keywords:** Cancer imaging, Cancer genomics, CNS cancer, Machine learning, Predictive medicine

## Abstract

Multi-omic data, i.e., clinical measures, radiomic, and genetic data, capture multi-faceted tumor characteristics, contributing to a comprehensive patient risk assessment. Here, we investigate the additive value and independent reproducibility of integrated diagnostics in prediction of overall survival (OS) in *isocitrate dehydrogenase* (IDH)-wildtype GBM patients, by combining conventional and deep learning methods. Conventional radiomics and deep learning features were extracted from pre-operative multi-parametric MRI of 516 GBM patients. Support vector machine (SVM) classifiers were trained on the radiomic features in the discovery cohort (n = 404) to categorize patient groups of high-risk (OS < 6 months) vs all, and low-risk (OS ≥ 18 months) vs all. The trained radiomic model was independently tested in the replication cohort (n = 112) and a patient-wise survival prediction index was produced. Multivariate Cox-PH models were generated for the replication cohort, first based on clinical measures solely, and then by layering on radiomics and molecular information. Evaluation of the high-risk and low-risk classifiers in the discovery/replication cohorts revealed area under the ROC curves (AUCs) of 0.78 (95% CI 0.70–0.85)/0.75 (95% CI 0.64–0.79) and 0.75 (95% CI 0.65–0.84)/0.63 (95% CI 0.52–0.71), respectively. Cox-PH modeling showed a concordance index of 0.65 (95% CI 0.6–0.7) for clinical data improving to 0.75 (95% CI 0.72–0.79) for the combination of all omics. This study signifies the value of integrated diagnostics for improved prediction of OS in GBM.

## Introduction

Glioblastoma (GBM) is the most common and aggressive primary brain neoplasm in adults with a dismal prognosis. Standard treatment consists of maximal safe surgical resection followed by radiation therapy concomitant with temozolomide (TMZ) chemotherapy, which yields a median overall survival (OS) of 14.6–16.7 months^1^. While GBM is nearly always fatal, there is overwhelming evidence that the prognosis of patients with GBM varies with patient age^2^; clinical features [ECOG score, addition of temozolomide, bilateral spread], performance score^2^, extent of surgical resection^2,3^, and molecular characteristics^4^, e.g., the mutational status of the *isocitrate dehydrogenase* (*IDH*) genes, and the methylation status of the *O*^6^*-methylguanine-DNA methyltransfera*se (*MGMT*) promoter^5^. However, accurate determination of *MGMT* methylation status is limited by several factors, such as inter-observer variability, assessment technique, and cutoff levels, imposing a challenge on multi-institutional clinical trials^6–8^. Furthermore, longer OS has been reported in a subset of patients with unmethylated *MGMT*, adding complexity to prediction of survival, and a personalized, precision approach to the patient^5^.

The advent of next generation sequencing (NGS) over a decade ago facilitated genomic medicine, with the tumor genetic information of each patient being increasingly integrated with molecularly-guided, patient-centered, diagnosis, prognosis, and treatment^9–11^. With the information about tumor genomics, druggable genetic targets can be identified that can potentially offer better patient outcomes^10^. GBM is known to harbor numerous genetic mutations, some of which give rise to genetic instability and additional mutations, and therefore, a heterogeneous response to treatment^12–14^. Clear correlation of genetic changes to outcomes in *IDH*-wildtype GBM have not been consistently demonstrated, although some reports suggest that particular mutations drive aggressive behavior^15–17^.

Over the past decade, MRI-based radiomics has emerged as a promising method in providing non-invasive and quantitative biomarkers that offer insights about the phenotypic characteristics of GBMs^18–23^. Radiomics can capture coarse and subtle characteristics of the tumor and its surroundings through quantification of the features of shape, distribution of intensity, texture, or higher-level statistics and combining them with machine learning (ML) approaches to build predictive models of a given disease endpoint, i.e. OS, recurrence, genomics, etc.^18,24,25^. In this study, we aimed to assess the potential of integrating multi-omics prognostic characteristics, including clinical measures, radiomics, *MGMT* methylation, and genomics, to predict OS in GBM patients. We postulate that multi-omics data integration can capture multi-faceted tumor characteristics at different scales, i.e., molecular (genomics and *MGMT* methylation), macroscopic (radiomics), and clinical, and therefore provide the clinicians with a comprehensive representation of the patient’s condition and risk, towards facilitating personalized treatment planning and more efficient clinical trial stratification. Here, we promote a radiomic signature of OS based on widely available pre-operative conventional multi-parametric MRI (mpMRI) scans by combining conventional and deep radiomics. Furthermore, we explore the incremental value of radiomics and genomics to the readily-available clinical measures.

## Materials and methods

### Study design

**Figure 1:**
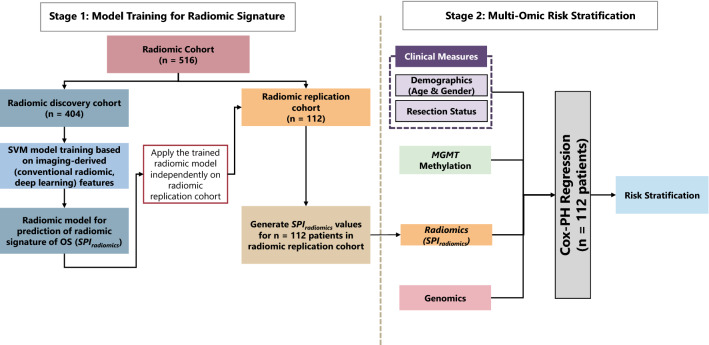
Multi-omic analysis method for risk stratification of patients with *IDH*-wildtype GBM tumors based on their radiomic signature (SPI_radiomics_), clinical measures [age, gender, and extent of resection (EOR)], and molecular information (*MGMT* methylation and genomics, obtained by next-generation sequencing (NGS) of the tumor samples).

This study design includes two stages of predictive modeling, as summarized in Fig. 1. In stage 1, we built a radiomic predictive model composed of two classifiers on the “radiomic discovery cohort” based only on imaging-derived (conventional radiomic and deep learning) features. The output of this stage was a single radiomic signature of OS per patient. The radiomic predictive model was independently applied to the features extracted for the patients in the “radiomic replication cohort” to calculate values for radiomic signature. The “radiomic replication” cohort was kept unseen during training for the radiomic predictive model (more details are provided in section “Data description”). This “radiomic replication cohort” was used as the cohort for model training–testing in stage 2. The vector of radiomic signature values computed for the patients in this replication cohort was then used as a predictor for risk stratification. In stage 2 or the risk stratification stage, multi-omic predictors, i.e., clinical measures, *MGMT* methylation, radiomic signature, and genomics for the “radiomic replication cohort” were integrated in a layered approach (details can be found in section “Stage 1: predictive modeling to generate radiomic signature of OS”). In what follows, we will describe our data and method in more details.

### Data description

All experimental protocols were approved by the institutional review board (IRB) of the University of Pennsylvania (UPenn) and all methods were carried out in accordance with relevant guidelines and regulations. Our retrospective study was compliant with the health insurance portability and accountability act (HIPAA) and obtained a waiver of informed consent from the IRB of UPenn. However, all patients had provided their informed consent to participate in research studies at the time of their imaging. We retrospectively collected a cohort of n = 617 adult patients, who underwent pre-operative mpMRI scanning followed by surgical resection at the Hospital of the University of Pennsylvania (HUP), between 2006 and 2018 and were histopathologically diagnosed with de novo GBM. Patients were excluded if: (1) their conventional MRI scans were incomplete (at least one of the conventional MRI sequences was missing); (2) *IDH* mutation was detected; (3) treated outside of HUP; or (4) not followed from the time of surgery until death to determine their OS. A cohort of n = 516 patients with newly diagnosed GBM were included, among which n = 112 had all required information for our final risk stratification model, i.e., demographics, extent of tumor resection (EOR), pre-operative mpMRI, *MGMT* promoter methylation, and genomic sequencing results. A cohort of n = 404 patients with available mpMRI were used as the “radiomic discovery cohort” for training our radiomic signature of OS. The cohort of n = 112 subjects with all available information was kept unseen and independent from the radiomic discovery cohort during the radiomic model training and considered as our “radiomic replication cohort”. Table 1 summarizes the characteristics of the included patients. All the patients had been followed at HUP until deceased.

**Table 1:** Characteristics of the included patients in the discovery and independent replication cohorts.

	Discovery cohort	Replication cohort
**Range of scan dates**	2006–2018	2012–2018
**Demographics**		
No. of patients	404	112
Median age (years)	63.9	65.7
Age range (years)	22.0–88.5	20.7–87.6
No. of females	176 (44%)	31 (28%)
**Extent of resection (no. of patients)**		
Near/Gross total resection	281 (69%)	57 (51%)
Partial resection or biopsy	123 (30%)	55 (49%)
**MGMT methylation status (no. of patients)**		
Methylated	43 (10%)	42 (37.5%)
Unmethylated	80 (20%)	70 (62.5%)
Indeterminate or not available (N/A)	281 (70%)	0
**Survival (months)**		
Median ± Std	12.1 ± 13.8	12.2 ± 11.0
No. of high-risk patients	114	28
No. of low-risk patients	109	28

#### MRI acquisition

All included patients had undergone pre-operative MRI acquisition on a 3 Tesla scanner (Siemens Magnetom Tim Trio, Erlangen, Germany) using a 12-channel phased array coil. Conventional MRI sequences included pre- (T1) and post-gadolinium contrast enhanced (T1-Gd) axial high-resolution three-dimensional (3D) magnetization-prepared rapid acquisition with gradient echo (MPRAGE) sequence, with Repetition Time (TR)/Echo Time (TE) = 1760/3.1, Flip angle (FA) = 15°, Field of View (FOV) = 187 × 250 mm^2^, Slice Thickness = 1 mm, Matrix Size = 192 × 256 × 192, resolution = 0.98 × 0.98 × 1; axial Turbo Spin-Echo T2-weighted (T2) imaging with: TR/TE = 5340/85, FA = 160°, FOV = 200 × 240 mm^2^, Slice Thickness = 3 mm, Matrix Size = 208 × 256 × 64, resolution = 0.94 × 0.94 × 3; axial Turbo Spin-Echo T2-weighted fluid-attenuated inversion recovery (T2-FLAIR) with TR/TE = 9420/141, FA = 170°, FOV = 180 × 240 mm^2^, Slice Thickness = 3 mm, Matrix Size = 192 × 256 × 60, resolution = 0.94 × 0.94 × 3.

#### Next-generation sequencing (NGS)

The resected tumor samples for the patients in this cohort were sequenced using one of the two in-house targeted NGS panels, as described below.

##### NGS Panel 1

Tumor samples for a cohort of n = 193 patients were evaluated with an in-house NGS panel. This custom AmpliSeq sequencing panel was designed targeting the CDS of 45 genes as well as *IDH1 p.R132*, *IDH2 p.R172*, and *BRAF p.V600*. Libraries were prepared from 10–40 ng FFPE DNA using the Ion AmpliSeq Library Kit 2.0 (ThermoFisher Scientific), multiplexed, and sequenced on an Ion Torrent S5 sequencer 540 chip targeting 3.4 M reads per sample for 1600 × average depth of coverage. Primary analysis and alignment of sequence reads was performed using Torrent Suite Software version 5.8 followed by SNV and indel calling using an Ion Reporter v5.10 single sample somatic variant calling workflow.

Ion Reporter variants were normalized using BCFtools followed by annotation with Annovar and filtering with BCFtools. Variants with quality scores < 30, read depth < 100, allele frequency < 0.05 and strand bias > 0.7 were filtered out, as were indels in homopolymer regions greater than 8 bp in length. Filtering on annotations retained only splice site and exonic (excluding synonymous) variants occurring in the gnomAD exome and genome databases with population frequency < 1%.

Potential sequencing artifacts were identified by manual curation of recurrent variants absent in gnomAD exomes (v2.1.1), COSMIC (v90), or CLINVAR (1/27/2020) as well as sequencing 10 HapMap samples obtained from the Coriell Institute, including NA12342 from the NIGMS Human Genetic Cell Repository, with the same panel. Variants classified as artifacts were also filtered out.

Target genes comprised: *TP53*, *PTEN*, *ATRX*, *EGFR*, *VEGF*, *PDGFRA*, *PIK3CA*, *PIK3R1*, *NF1*, *PDL1*, *CTLA4, HIF1A, MDM4, RB1, STAT1, CD70, CIC, FUBP1, CDK4, ACADL, TRIM26, SMAD1, ARID2, CDKN1B, CREBZF, DNMT3A, EPHB2, ERRFI1, FGFR2, GIGYF2, KDR, KRAS, MAP3K1, MET, NIPBL, NOTCH2, NRAS, NTRK1, PTBP1, PTPN11, SETD2, SMARCB1, TP63, WRN*. The hotspots included *IDH1 p.R132*, *IDH2 p.R172*, *BRAF p.V600, H3F3A p.K28*, *TERT* (chr5:1295151–1295315). However, *H3F3A* were effectively not covered due to presence of pseudogene, that was ignored by variant caller, and *TERT* showed poor amplicon performance and excluded.

##### NGS Panel 2

We collected the genomic data for n = 181 patients that had their tumors sequenced in another panel of 153 actionable and prognostic genes for sequencing solid tumors, implemented and validated at our institution for clinical assessment of the resected tumors. With the Agilent Haloplex design, the panel provides a full coverage of all included genes. A complete description of this panel and the in-house data processing bioinformatics pipeline has been previously documented^26^.

##### Final genomic cohort

A total of 27 genes were included in both panels and therefore, used for our integrated risk stratification approach for n = 374 patients: *ARID2*, *ATRX*, *BRAF*, *CDKN2A*, *CIC*, *DNMT3A*, *EGFR*, *FGFR2*, *FUBP1*, *IDH1*, *IDH2*, *KDR*, *KRAS*, *MDM4*, *MET*, *NF1*, *NOTCH2*, *NTRK1*, *PDGFRA*, *PIK3CA*, *PIK3R1*, *PTEN*, *PTPN11*, *RB1*, *SETD2*, *SMARCB1*, *TP53*. We excluded patients with mutations in *IDH1* or *IDH2*. The final genomic cohort comprised of n = 112 *IDH*-wildtype patients with available OS data who had all other information, i.e., clinical measures, *MGMT* methylation status, and mpMRI available.

#### DNA methylation profiling

Genomic DNA was extracted from tumor samples, underwent bisulfite conversion, and was then amplified with primers which target DMR2 of the MGMT (O-6-methylguanine-DNA methyltransferase, NM_002412) promoter, including 4 CpG sites (chr10:131265519–131265537; hg19 Assembly). The PCR product was evaluated using pyrosequencing (PyroMark Q24; Qiagen, Hilden, Germany) to determine the percent methylation across the 4 CpG sites. A sample was called methylated if both the mean and median percentage methylation calculated over the 4 CpG sites was ≥ 10%^26^ and considered low positive when mean and median level of DNA methylation are either relatively low (i.e., above the limit of detection but below 10%) or highly variable across the 4 CpG sites. A not detected result was determined when the mean and median percent methylation across the 4 CpG sites were below the limit of detection (4.5%).

### Stage 1: predictive modeling to generate radiomic signature of OS

#### Image pre-processing

The mpMRI volumes were preprocessed using Cancer Imaging Phenomics Toolkit open-source software (CaPTk, https://www.cbica.upenn.edu/captk)^27,28^. For each of the patients, the raw DICOM images were converted to NIfTI format, reoriented to the left-posterior-superior coordinate system, then rigidly co-registered and resampled to a spatial resolution of 1 × 1 × 1 mm^3^ based on the SRI atlas^29^. All image registrations were performed using the Greedy tool (https://github.com/pyushkevich/greedy)^30^. The images were then skull-stripped using the Brain Mask Generator (BrainMaGe)^31^ and corrected for intensity inhomogeneities and noise. Image intensities were scaled to the range of (0, 255) after removing the outlier pixels that did not fall into 99.9% percentile of the image histogram. Automatic segmentation of tumoral subregions, including the enhancing tumor (ET), necrotic tumor core (NC), and peritumoral edematous/infiltrated tissue (ED), was performed with the deep learning brain tumor segmentation module of CaPTk v.1.8.1^27,28^, that is based on DeepMedic^32^ and revised when necessary.

#### Conventional radiomic features

The pre-processed mpMRI scans and segmentations of tumor subregions were passed through the feature extraction panel of CaPTk to calculate radiomic features automatically, for each of the imaging scans (T1, T1-Gd, T2, T2-FLAIR) and based on each tumoral subregion, i.e., ET, NC, ED. A total of 1032 radiomic features were extracted with feature categories of first-order intensity-based statistics, histogram, volumetric, gray-level co-occurrence matrix (GLCM), gray-level run length matrix (GLRLM), gray-level size-zone matrix (GLSZM), neighborhood gray-tone difference matrix (NGTDM)^33^, and Collage features^34^. All extracted features were normalized using z-scoring before further analyses.

#### Deep radiomic features

**Figure 2:**
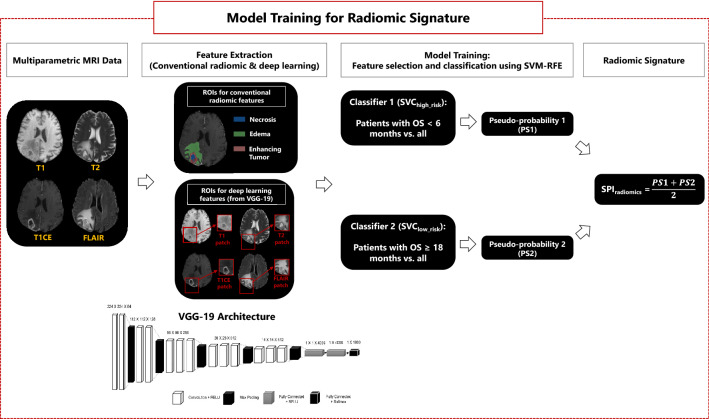
An illustration of model training process for predicting the radiomic signature of overall survival (OS). After preprocessing of multiparametric MRI scans, conventional radiomic features are extracted from the segmented tumorous subregions, i.e., necrosis (NC), edema (ED), and enhancing tumor (ET). Deep learning features are derived from the whole tumor region (union of NC, ED, and ET subregions) using VGG-19 network architecture. Two binary classifiers are built: SVC_high_risk_ to discriminate the short survivor patients from other patients, and SVC_low_risk_ to differentiate the long survivor patients from the rest of the patients. The output of each classifier, i.e., pseudo-probability1 or pseudo-probability2, are combined to generate SPI_radiomics_, which represents the radiomic signature of OS for the patients.

We further extracted a set of deep radiomic features obtained from a pre-trained deep learning model using transfer learning, i.e., VGG-19 with 16 convolutional neural networks including rectified linear unit (RELU) pooled with three fully connected layers. This model has been pre-trained on a natural image dataset ImageNet (http://www.image-net.org) and shown excellent performance in image classification tasks^35,36^. The architecture of VGG-19 is illustrated in Fig. 2. We selected a bounding box (or a patch) encompassing the entire tumor, i.e., the union of ET, NC, and ED, on mpMRI (T1, T1-Gd, T2, T2-FLAIR) scans, which were then resized to a dimension of 224 × 224 using cubic interpolation and fed as inputs to the deep networks. Front propagation with pre-trained weights was performed for initialization and extracted 8192 features from the first two fully connected layers.

#### Predictive modeling

For radiomics analysis, as illustrated in Fig. 2, conventional and deep radiomic features that were extracted from the radiomic discovery cohort (n = 404) were used for predictive modeling using machine learning. We built two different binary support vector machine (SVM) classifiers with recursive feature elimination (RFE-SVM). One SVM classifier (SVC_high_risk_) was designed to differentiate the high-risk patients, who survived less than 6 months, from the rest of the subjects. The second SVM classifier (SVC_low_risk_) was trained to discriminate the lower-risk patients, who survived beyond 18 months, from all other patients.

After normalizing the features using z-scoring (subtraction of the mean and division with the standard deviation), we removed all features with small variations as determined by the mean absolute deviation (MAD). The adopted RFE-SVM method searches for the optimal feature subset by fitting the SVM model to the feature space and removing the weakest features until the best performing feature subset is achieved. The classifiers were trained with 5-fold nested cross validation (nested-CV) to ensure generalizability. For nested-CV, the data was divided into five folds (for external cross-validation), and each external fold was further divided into another five folds (for internal cross-validation). In the internal folds, feature selection and SVM hyperparameter tuning were performed. In each external fold, with the feature subset and hyperparameter values that were selected in the internal folds, SVMs were trained based on four out of five folds of the data and validated on the remaining fold. From the five SVM models trained on the external folds, the one with the best validation accuracy was chosen to be applied to the independent replication data.

The outputs of the two binary classifiers, i.e., SVC_high_risk_ and SVC_low_risk_, were distances of the data points to the SVM hyperplanes. A sigmoid function was fitted to the outputs of each classifier to generate indices representing pseudo-probabilities of data points belonging to a class. The indices from the two classifiers were then averaged to create a survival prediction index (SPI_radiomics_). A higher value of SPI_radiomics_ corresponds to a higher OS (i.e., a lower-risk), while a lower value of SPI_radiomics_ associates with a lower OS (i.e., a higher-risk). The resulting model, with the hyperparameter values optimized through 5-fold nested CV based on the discovery cohort (n = 404), was tested on the selected features in the model calculated for our independent replication cohort (n = 112) and SPI_radiomics_ values were generated for the patients in this cohort.

We designed our radiomics predictive model to be compatible with clinical scenarios, where short survivor/higher risk patients may be prescribed with a supra-total resection for their surgery and dose escalation in radiation therapy. On the contrary, long-survivor/lower risk patients may receive aggressive local treatments without supra-total resection^21^. Therefore, we have regulated our predictive model to characterize the two extremes that can affect the clinical decision making about the appropriate management recommendation of these patients.

### Stage 2: multi-omic risk stratification

We employed six Cox proportional hazards (Cox-PH) models based on multi-omics predictors, i.e., clinical measures, *MGMT* methylation, radiomic signature, and genomics to risk stratify the patients in terms of their predicted OS. We trained Cox-PH models for the following different levels of data integration:

1. Clinical measures, including demographics (age, gender), and EOR.
2. Clinical measures and *MGMT* methylation status.
3. Clinical measures and radiomic signature (i.e., SPI_radiomics_).
4. Clinical measures, *MGMT* methylation, and radiomic signature.
5. Clinical measures, *MGMT* methylation, and genomics.
6. Clinical measures, *MGMT* methylation, radiomic signature, and genomics.

The multi-omics risk assessment method is indicated in Fig. 1.

#### Dimensionality reduction of genomic variables

Genomic data included 27 genes (variables) with categorical values for mutated and wildtype labels for n = 112 patients. We considered a Cox-PH model with least absolute shrinkage and selection operator (LASSO) penalty, to reduce the dimensionality of the genomic variables by identifying a subset of variables that are predictive of OS^37^. The LASSO estimator performs feature selection by shrinking the number of regression coefficients to zero. This degree of shrinkage is controlled by the parameter λ, which is typically found through CV as the value which maximizes the partial likelihood. Because the random partitions in CV can produce highly variable results, both in terms of the selected variables and the model performance, we used two nested CV loops^38^: the inner loop selected the optimal λ, and the outer loop assessed out-of-sample performance. In each outer loop, the data was randomly partitioned into outer training (n = 75) and outer test (n = 37) sets. In the inner loop, 3-fold CV on the outer training set was repeated 100 times to determine the value of λ_min_ that minimized average deviance over the repetitions. Prediction performance of the model fit using λ_min_ was then assessed on the outer test set. The outer loop was repeated 100 times and the value of λ_final_ with highest c-index in the testing sets was selected as the parameter in the final LASSO regression. The output was a list of selected genomic variables that were incorporated with other omic data.

#### Multi-omic data integration for risk startification

Each of the 6 models were fit on the same set of subjects (n = 112) that had complete clinical, *MGMT* methylation, radiomic, and genomics data. We considered maximum partial likelihood estimator (MPLE), as an estimator of the regression coefficients for variables in the models. For all models, prediction accuracy was assessed using the concordance (c-) index, which ranges from 0 (worse fit) to 1 (better fit). We further gauged model performance based on the Integrated Brier Score (IBS) ranging from 0 (better) to 1 (worse) as compared to a reference model. Statistical analyses were performed in R version 3.6.0 (R Foundation for Statistical Computing, Vienna, Austria). There was no censoring, as the event time was observed for all individuals. This study did not have any missing data.

## Results

### Stage 1 modeling: radiomics signature of overall survival

**Figure 3:**
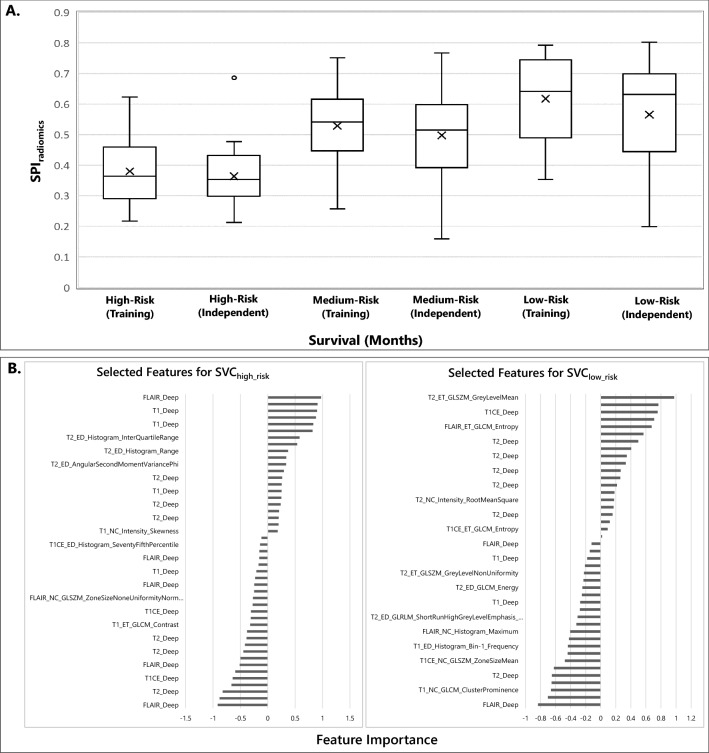
(**A**) SPI_radiomics_ versus survival for high-risk (< 6 months), medium-risk (≥ 6, < 18 months), and low-risk (≥ 18 months) patient groups for the training and independent cohorts. Survival was maximum amongst the patients classified as the long survivors or low-risk, minimum amongst those classified to be short survivors or high-risk. (**B**) The selected features (n = 47) for the high-risk classifier, i.e., SVC_high_risk_ (left), and the selected features (n = 44) for the low-risk classifier, i.e., SVC_low_risk_ (right). The y-axis in this plot represents the selected features and the x-axis denotes the importance of features in the model.

Radiomic analysis demonstrated an AUC of 0.78 (95% CI 0.70–0.85) for the SVC_high_risk_ (6-month) and 0.75 (95% CI 0.65–0.84) for the SVC_low_risk_ (18-month) classifier, in the training cohort. In the independent replication cohort, the classification performance of SVC_high_risk_ and SVC_low_risk_ classifiers in terms of AUC were 0.75 (95% CI 0.64–0.79) and 0.63 (95% CI 0.52–0.71), respectively. Figure 3A presents box plots of SPI_radiomics_ vs actual survival in months (greater value of SPI_radiomics_ predicts more prolonged survival, and lower SPI predicts shorter survival) for the independent and training cohort. A summary of the selected radiomic features for the SVC_high_risk_ and SVC_low_risk_ classifiers can be found in Fig. 3B, suggesting that the majority of the selected features are among deep features.

### Stage 2 modeling: risk stratification

The Cox-PH model using clinical variables (i.e., age, gender, and EOR), showed a c-index of 0.65 (95% CI 0.60–0.70) with the largest coefficient for EOR with HR = 0.43 (95% CI 0.29–0.64; p < 0.0001), indicating that gross total or near total resection of the tumor was associated with longer OS. Adding *MGMT* methylation to the clinical variables slightly improved the c-index of the created Cox model to 0.67 (0.62–0.72). In this model, the significant predictors were *MGMT* methylation with HR = 0.56 (95% CI 0.36–0.87; p = 0.01), and EOR with HR = 0.44 (95% CI 0.29–0.65; p < 0.0001). These results suggest that *MGMT*-methylated tumors and larger extent of resection are associated with a lower hazard of short survival.

The model including clinical and SPI_radiomics_ covariates returned an improved c-index = 0.70 (95% CI 0.65–0.75), compared to the clinical model. This model showed the largest negative coefficient for SPI_radiomics_ with HR = 0.13 (95% CI 0.04–0.45; p = 0.001), implying that the higher SPI_radiomics_ was associated with lower hazard of short survival. After adding *MGMT* methylation status to the clinical and SPI_radiomics_ variables, c-index improved to 0.72 (95% CI 0.68–0.77). SPI_radiomics_ showed a low HR of 0.07 (95% CI 0.02–0.27; p = 0.0001).

The genomic variables were penalized using LASSO method in a Cox-PH model including genomic mutations and the selected genes were *EGFR*, *MET*, *NOTCH2*, *PDGFRA*, and *RB1*. The Cox-PH model including clinical, *MGMT* methylation status, and genomic variables showed a c-index of 0.70 (95% CI 0.66–0.75), performing similarly to the model composed of clinical and SPI_radiomics_ covariates. Significant predictors of OS in this model were patient’s EOR (HR = 0.35, 95% CI 0.22–0.54; p < 0.0001), Age (HR = 1.03, 95% CI 1.01–1.05; p = 0.002), *MGMT* methylation status (HR = 0.53, 95% CI 0.34–0.81; p = 0.004), *RB1* (HR = 0.38, 95% CI 0.19–0.76; p = 0.006), and *NOTCH2* (HR = 0.2, 95% CI 0.06–0.71; p = 0.01).

**Figure 4:**
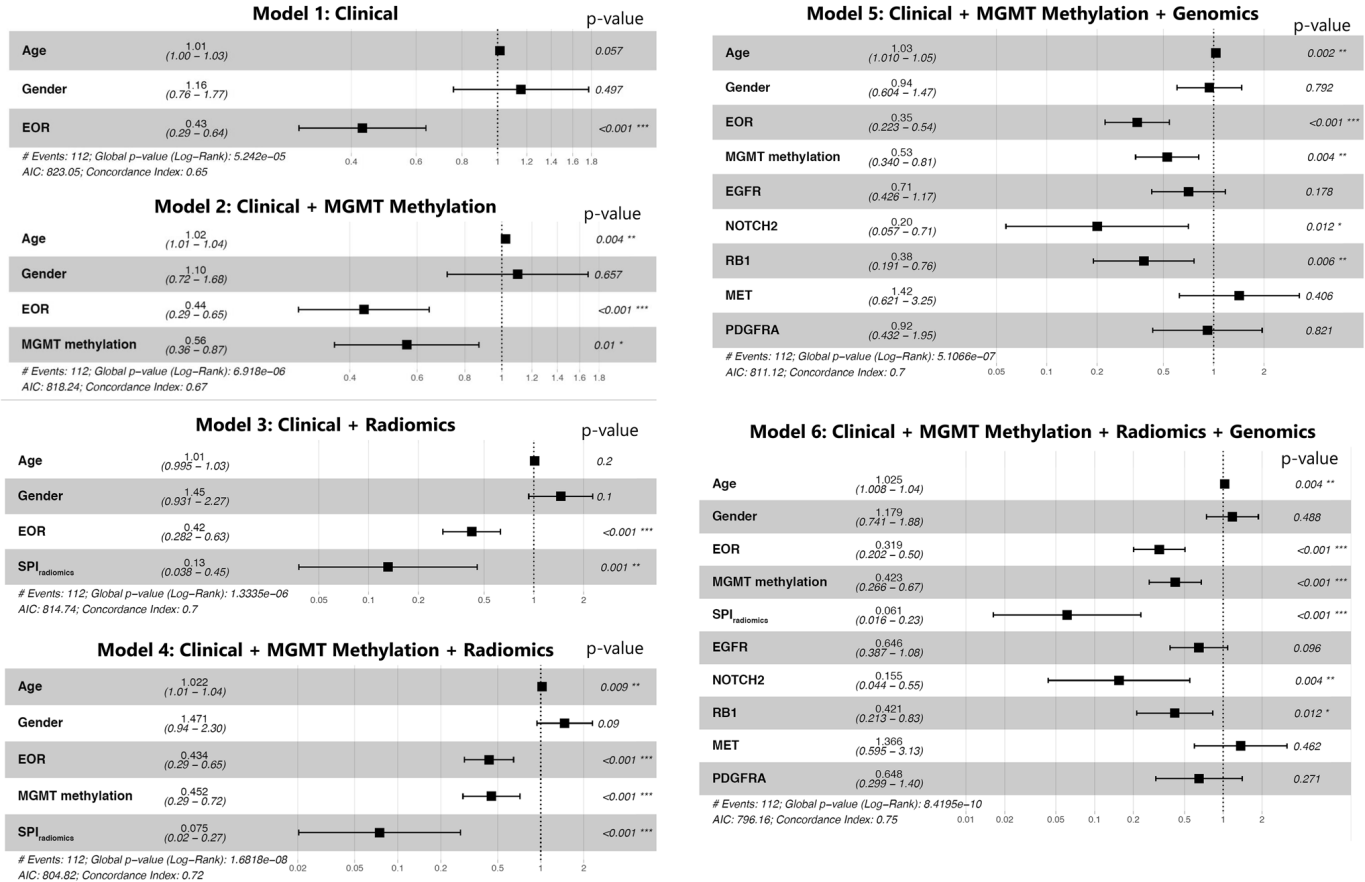
Forest plots for the six presented Cox-PH models, demonstrating the association between each of the covariates and survival. In this plot, x-axis presents hazard ratios (HR) for the covariates in each model (the value of HR and confidence intervals for the covariates are provided on the plot as well).

Finally, the last model integrating all data sources, i.e., clinical, *MGMT* methylation, SPI_radiomics_, and genomic, showed an improved c-index = 0.75 (95% CI 0.71–0.78), where significant covariates included EOR (HR = 0.32, 95% CI 0.20–0. 50; p < 0.0001), SPI_radiomics_ (HR = 0.06, 95% CI 0.02–0.23; p < 0.0001), *MGMT* methylation status (HR = 0.42, 95% CI 0.27–0.67; p = 0.0003), Age (HR = 1.03, 95% CI 1.01–1.04; p = 0.004), *NOTCH2* (HR = 0.15, 95% CI 0.04–0.55; p = 0.004), and *RB1* (HR = 0.42, 95% CI 0.21–0.83; p = 0.01). Together, these results suggest that our fully integrated (i.e., the multi-omics) model containing all derived features is the most predictive of survival time. An illustration of forest plots of the covariates in each of the models and their hazard ratios is provided in Fig. 4.

Table 2 summarizes the performances of the 6 designed Cox-PH models. The results in this table show an increased c-index and reduction in IBS error, suggesting an improvement in risk stratification when a further layer of information is added to the integrated prognostic models. Including *MGMT* methylation with the clinical variables yielded a 14.2% reduction in prediction error with respect to the reference IBS, integrating radiomics with clinical data resulted in 15% decrease, a combination of clinical, *MGMT* methylation, and radiomics achieved 19.4% decrease in prediction error, similar to an integration of clinical, *MGMT* methylation, and genetics with 19.8% decline in IBS. The highest performance was achieved with the model composed of clinical, *MGMT* methylation, radiomics, and genomics, as evidenced by the c-index of 0.75 and IBS reduction of 24.8%.

**Table 2:** Performance metrics for the Cox-PH models.

Model	c-index (95% CI)	IBS	IBS difference* (%)
Model 1: Clinical	0.65 (0.6, 0.7)	0.101	− 10.3
Model 2: Clinical + *MGMT* methylation	0.67 (0.62, 0.72)	0.097	− 14.2
Model 3: Clinical + Radiomics	0.70 (0.65, 0.75)	0.096	− 15
Model 4: Clinical + *MGMT* methylation + Radiomics	0.72 (0.68, 0.77)	0.091	− 19.4
Model 5: Clinical + *MGMT* methylation + Genomics	0.70 (0.66, 0.75)	0.091	− 20.3
Model 6: Clinical + *MGMT* methylation + Radiomics + Genomics	0.75 (0.72, 0.79)	0.086	− 24.8

^†^*CI* confidence interval.

*The difference of IBS calculated from the Cox model compared to the reference IBS, which is equal to 0.113 in our study.

**Figure 5:**
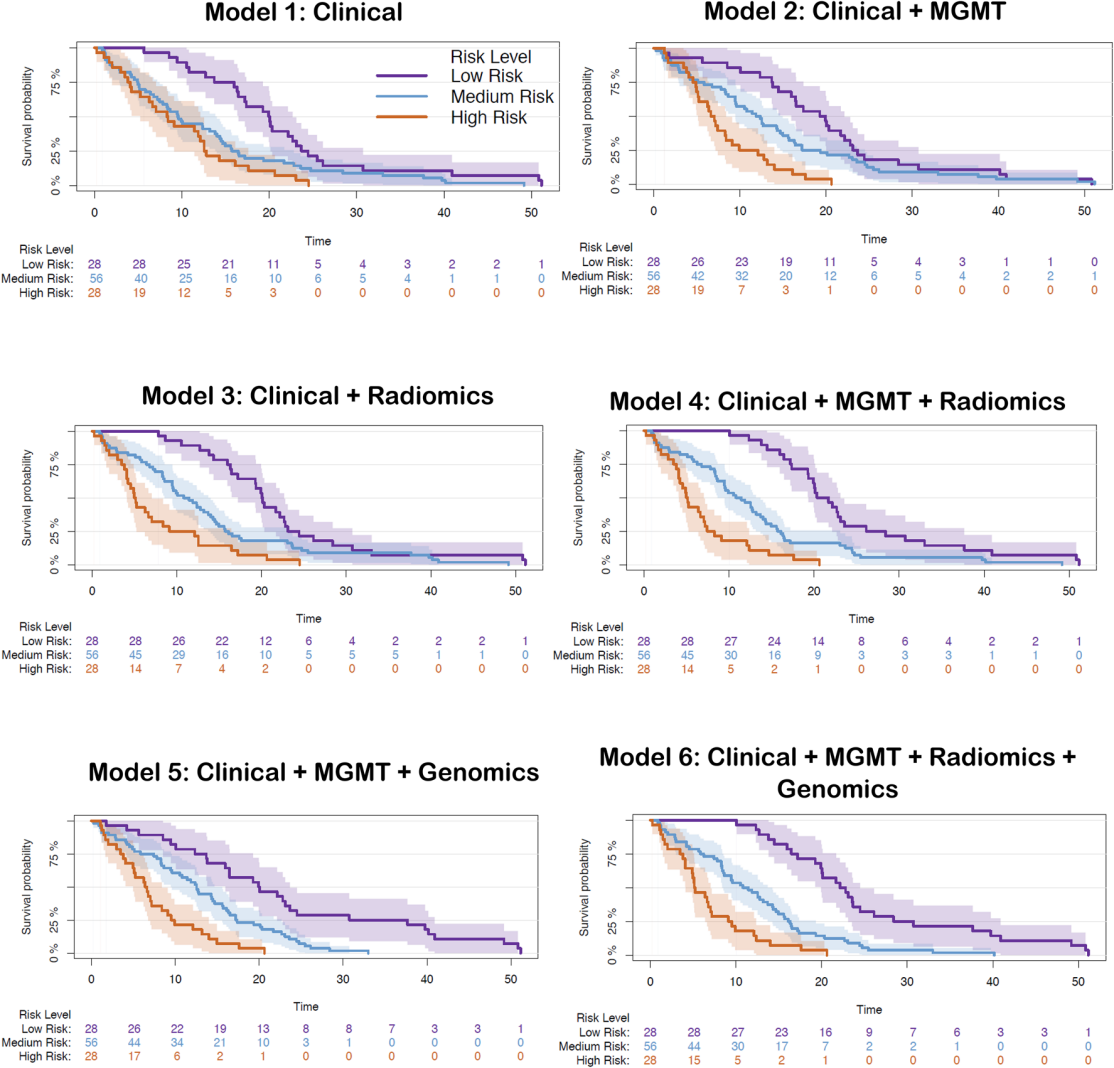
Risk stratification based on overall survival for the patients in our cohort (n = 112) using the six Cox-PH models including different layers of information. For illustration purposes, we have displayed low, medium, and high levels of survival probability. The survival curves are illustrated with their 95% confidence intervals. Log-rank test was performed to test for differences between the survival functions of the low, medium, and high risk groups predicted by the Cox model. The p-values obtained using this test for Model 1 (Clinical): p = 0.00016; Model 2 (Clinical + *MGMT*): p = 1e−5; Model 3 (Clinical + Radiomics): p < 1e−5; Model 4 (Clinical + *MGMT* + Radiomics): p < 1e−5; Model 5 (Clinical + *MGMT* + Genomics): p < 1e−5; Model 6 (Clinical + *MGMT* + Genomics + Radiomics): p < 1e−5.

Survival curves for high, medium, and low risk groups are depicted in Fig. 5, suggesting a synergistic interaction of different sources of information in stratification of high, medium, and low risk patients. As apparent from the survival curves, with the addition of a layer of information, discrimination of the risk groups improves. Model 2 with a combination of clinical and *MGMT* methylation data can better differentiate the low from medium risk groups compared with model 1 with only clinical information. The incremental value of radiomics to this combination can be observed from the curves in model 4, where the three risk groups are distinctly stratified. Genomics build on this discriminative potential of clinical, *MGMT*, and radiomics variables in model 6 and the risk groups become further differentiated. The results for model 3, i.e., clinical and radiomics, show that in absence of any sequencing, including *MGMT* methylation and genomics, radiomics signature created based on pre-operative MRI, can provide an added value to the clinical predictors of OS and provide a distinct stratification of the risk groups. The similarity in performance of models 4 and 5 (as suggested by their c-index of 0.70 in Table 2) is further evidenced from their corresponding survival curves.

## Discussion

This study introduces a multivariate integration of multi-omics data for risk stratification of patients with newly diagnosed, treatment-naïve *IDH*-wildtype GBM. We used information collected from several sources of data to build multiple models, each with different layers of prognostic information for disease stratification. Starting from the base model including the least available information for the patients with GBM, i.e., clinical variables of age, gender, and extent of resection, we showed incremental value of radiomics, *MGMT* methylation, and genomic data obtained by NGS sequencing of the tumor samples, resulting in a multi-omics model with superior performance.

Despite marked variation in treatment responsiveness and outcomes, nearly all patients with newly diagnosed GBM still receive the same standard radiation and temozolomide-based therapy, underscoring a lack of personalized treatment in GBM relative to many other solid tumors^39^. Moreover, interpatient heterogenetiy in both tumor biology and clinical characteristics has made it difficult to accurately determine the efficacy of experimental treatments, particularly in newly diagnosed GBM trials with progression-free survival (PFS) or OS endpoints^40,41^. Our findings suggest that multi-omics or multi-scale fusion of data can provide a comprehensive portrayal of tumor biology and the patient’s likely OS. If validated in a larger, multi-center study, this survival prediction tool has the potential for widespread clinical and research use, allowing for more efficient clinical trial design in the newly diagnosed setting which may lead to more personalized treatments for the patients.

In the present study, the image-based predictive model of OS builds upon and reinforces prior studies^20,23^ by employing an advanced computational methodology based on high-throughput data, i.e., conventional and deep radiomic features. We showed generalizability of our non-invasive radiomic prognostic approach to unseen data, evidenced by the classification performance of our SVM models. An important strength of our proposed signature is that it is derived from pre-operative conventional MRI scans which are acquired as a part of standard-of-care diagnosis of patients with GBM. In both of our SVM classifiers, i.e., high-risk and low-risk models (SVC_high_risk_ and SVC_low_risk_, respectively), most of the selected features were among deep features derived from T2 and T2-FLAIR scans, emphasizing the significance of these features in quantifying higher-order patterns. GBM tumors exhibit spatial heterogeneity arising from variation in cellular density, vascularization, and necrosis across their area^42^, and deep learning features enable detection of phenotypic characteristics within pre-operative images that may not be captured comprehensively by only conventional radiomic features.

The survival prediction index (SPI_radiomics_) was fused with other omics data for risk stratification of patients with GBM. As the results suggest, SPI_radiomics_ provided additive value over the clinical and *MGMT* data for risk assessment, improved the concordance index, and provided more distinctive separation of the low, medium, and high-risk groups. Genetics further improved risk stratification in synergy with other omics. Added value of a radiomics signature to clinical and *MGMT* methylation in prediction of OS and PFS in patients with GBM has been reported in only a few studies with smaller cohorts^22,43^. The current study contributes to the improvement of the interpretability, predictive applicability and computational efficiency of existing machine learning algorithms, thereby increasing the precision of population-based registries and validate current and future prediction tools.

A few studies have explored the contributions of imaging and genomics features to prediction of OS in patients with GBM. In a study, higher values of relative cerebral blood volume in the nonenhancing region of the tumor (rCBV_NER_) in the *EGFR*-wildtype GBM patients was suggested to be associated with poorer survival^44^. The maximum value of this rCBV within the enhancing tumor (rCBV_max_) was also found to be a strong predictor of OS along with the Verhaak molecular classification^45^. Another study analyzed and indicated the potential of incorporating radiomic features with clinical measures and *MGMT* methylation using a penalized Cox regression model on 181 patients^22^. On a cohort of 200 patients, a study was performed on integration of radiomic features with clinical factors, genomics, transcriptomics, and proteomics to achieve a classification performance (in terms of AUC) of 78.2%^46^.

This paper presents several contributions in addition to the current literature. First, it presents a comprehensive study of prognosis of OS leveraging machine learning-based integration of clinical, imaging, and genomic data drawing from a cohort totaling 516 patients, including an independent replication dataset. To our knowledge, this is the first study of this size to explore the synergistic value of genetic, imaging and clinical predictive factors. Second, it demonstrated that a combination of deep learning and conventional radiomics produces a strong predictive panel of radiomic features for OS using standard, routinely acquired MRI scans. Third, it demonstrates the relative value of each omics in the context of additive contribution of clinical, imaging, and genetic variables. Critically, proper integration of all these measures via machine learning produced high predictive value on an independent test cohort of 112 patients, thereby further bolstering our confidence in the reproducibility and clinical value of these emerging AI-based integrated precision diagnostic indices.In studies including only genomics, genetic classification of *IDH*-wildtype GBM^15^ has not yielded prognostic information, although a recent transcriptomic classification based on developmental and metabolic axes has dissected apart subclasses of GBM with prognostic significance and contrasting metabolic vulnerabilities^47^. Our approach revealed two genes, *RB1* (retinoblastoma tumor suppressor protein) and *NOTCH2*, previously studied for their role in oncogenesis having significant associations with survival. In contrast, variants in other studied genes, including EGFR, did not show a significant association with survival. *RB1* is involved in the cell cycling pathway, allowing progression from G1 to S phase, activated by phosphorylation and regulated by *CDK4/CK6* protein kinases^48,49^. Alterations affecting the function of the retinoblastoma pathway were detected in 78.9% of glioblastomas studied in TCGA, with a minority of these alterations including direct mutation of *RB1*^12^. Identifying these *RB1* mutations is relevant to treatment, because tumors harboring inactivation of *RB1* are unlikely to respond to *CDK4/6* inhibitors, which have been developed and are being investigated as glioma therapy^48,50^. The fact that although a majority of GBM harbor alterations in the *Rb* pathway, the subset harboring *RB1* variants specifically are associated with a better prognosis will be relevant to the interpretation of trials of *CDK4/6* inhibitors and other *Rb* pathway-targeted therapies.

The significance of the *Notch2* variants is less clear, given the complicated biology of the Notch signaling pathway. This signaling pathway, discovered in *Drosophila* nearly a century ago, is known to be involved in numerous cellular processes, including signaling in solid tumors^51^. Changes in expression levels of *Notch2* and changes in the signaling pathway have been found in glioblastoma, specifically the glioma stem cell population^52^. The finding of a significant association of *Notch2* variants with better survival relative to the full GBM cohort is unexpected and intriguing. Further investigation of the effect of the specific mutations is necessary to understand the downstream consequences on canonical and noncanonical *Notch* pathways.

A limitation of our study is the single institutional data analysis. To achieve a generalizable method, it would be beneficial to explore data collected from multiple institutions. Furthermore, the sample size for stage 2 (risk stratification) (n = 112) was a potential limitation, mainly due to the unavailability of all omics information for all patients in our cohort. In particular, as broader molecular datasets become available on more patients, the prognostic implications of the molecular changes in the context of other omics may become clearer. Future studies would benefit from multi-institutional, prospective, and larger study population, a goal which the ReSPOND consortium is aiming to achieve^53^.

In conclusion, the present study fuses multiple omics data, namely clinical information, *MGMT* methylation, radiomics, and genetics, to accurately model clinical outcome in patients with newly diagnosed GBM*.* Accurate stratification of risk groups may facilitate improved patient management through personal optimization of treatment decisions, as well as effective risk stratification of patients for newly diagnosed GBM clinical trials.
